# A Narrative Review on Therapeutic Potentials of *Watercress* in Human Disorders

**DOI:** 10.1155/2021/5516450

**Published:** 2021-05-07

**Authors:** Esmaeel Panahi Kokhdan, Hadi Khodabandehloo, Hossein Ghahremani, Amir Hossein Doustimotlagh

**Affiliations:** ^1^Medicinal Plants Research Center, Yasuj University of Medical Sciences, Yasuj, Iran; ^2^Department of Clinical Biochemistry, School of Medicine, Zanjan University of Medical Sciences, Zanjan, Iran; ^3^Department of Clinical Biochemistry, School of Medicine, Shahid Beheshti University of Medical Sciences, Tehran, Iran; ^4^Department of Clinical Biochemistry, Faculty of Medicine, Yasuj University of Medical Sciences, Yasuj, Iran

## Abstract

*Watercress* (*WC*) is an aquatic vegetable that belongs to the Brassicaceae family, and it often grows near water. In traditional medicine, *WC* is a known remedy for hypercholesterolemia, hyperglycemia, hypertension, arthritis, bronchitis, diuresis, odontalgia, and scurvy. It also acts as an antiestrogenic and can be used as a nutritional supplement. It has been reported that these therapeutic effects are due to primary metabolites such as isothiocyanates, glucosinolates, polyphenols (flavonoids, phenolic acids, and proanthocyanidins), vitamins (B_1_, B_2_, B_3_, B_6_, E, and C), terpenes (including carotenoids), and bioelements which exist in this plant. Many pharmacological studies confirm the antioxidant, antibacterial, anticancer, antipsoriatic, anti-inflammatory, cardioprotective, renoprotective, hepatoprotective, and antigenotoxicity effects of *WC*. The consumption of *WC* extract can be useful in reducing the complications of hypercholesterolemia and hyperglycemia. Furthermore, the extract of *WC* could markedly augment the antioxidant enzymes such as superoxide dismutase and catalase activity. Interestingly, consumption of food rich in polyphenols such as *WC* extract can help reduce oxidative stress, DNA damage, and cancer susceptibility. Several studies also showed that *WC* extract significantly reduced liver injury as a result of cholestatic hepatic injury, gamma radiation, arsenic, and acetaminophen-induced hepatotoxicity. In this review, the researchers focus on the phytochemical and biochemical characterizations of *WC* and its therapeutic effects in the treatment of human diseases.

## 1. Introduction

**Figure 1:**
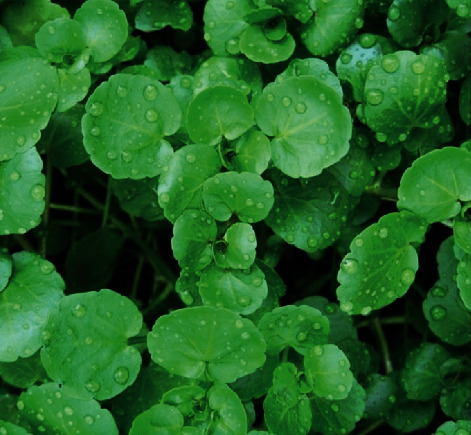
*Watercress* (*Nasturtium officinale)*.

*Watercress* (*Nasturtium officinale* L.) is an aquatic perennial leafy vegetable that is a member of the Brassicaceae family and it is usually found in and/or around water (Figure 1). Although this plant is native to western Asia, Europe, India, and Africa, its cultivation is not limited to these regions as it can grow anywhere on earth. Farmers used to plant *WC* in ponds, lakes, and slow-moving water like streams, rivers, and canals with moderately alkaline conditions. *WC* has Latin equivalents such as *Rorippa nasturtium aquaticum*, *Nasturtium aquaticum*, and *Rorippa officinalis*. As a plant, *WC* has recognized nutritional values but it seems that its biological properties suffer undue neglect [1, 2].

The traditional medicine of Azerbaijan, Iran, Morocco, and Mauritius western Asia, India, Europe, and Africa has made extensive uses of *WC* [1, 2]. Turkish folk medicine used *WC* as a drug for relieving abdominal pain. They also eat it as a vegetable and some even add it to their salads [3]. Iranian traditional medicine administered it as an antidiabetic agent, and Iranian people consumed it as an ingredient in their juices, salads, or other dishes [4]. Today, manufacturers have recognized the potential values of *WC*, so they use it to produce healthy foods and cosmetic products [1]. *Watercress* is consumed as fresh as possible, and it can be added to soups and other dishes. As a home remedy, the leaves of *WC* can be used as diuretic, depurative, expectorant, odontalgic, and hypoglycemic agents [5]. Moreover, *WC* shows exquisite resilience in polluted soils and in polluted water that contains heavy metals [6]. The crude extract of *WC* indicates high levels of antioxidant capacity in vitro. This extract also can inhibit the three stages of carcinogenesis including initiation, proliferation, and metastasis in cancer cell models that are present in vitro [7].

In traditional medicine, *WC* is an effective drug in treating hypercholesterolemia, hypertension, hyperglycemia, asthma, arthritis, cough, catarrh of the respiratory tract, bronchitis, diuresis, influenza, scurvy, and tuberculosis. It is a known appetizer for digestion complaints and can be used as a nutritional supplement. It is also famous for its expectorant, odontalgic, and antiestrogenic activity [1, 8]. In Germany, the *WC* is an FDA-approved plant that can be used in phytotherapy, rhinitis, and homeopathic medicines [1]. These therapeutic effects can be ascribed to primary metabolites such as glucosinolates, polyphenols (flavonoids, phenolic acids, and proanthocyanidins), isothiocyanates, vitamins (B_1_, B_2_, B_3_, B_6_, E, and C), terpenes (including carotenoids), and bioelements that exist in the plant. The most significant group of secondary metabolites that exist in the plant are glucosinolates [1, 9]. It has been found that *WC* leaves contain 14 phenolic compounds, i.e., gallic acid, coumaric acid derivatives, ferulic acid, apigenin, proanthocyanidin B1, sinapic acid, hydroxybenzoic acid, coumaric acid, caffeoyl malic acid, caftaric acid, kaempferol, and glucosides of quercetin [10]. Many pharmacological studies have confirmed the antioxidant, antibacterial, anticancer, antipsoriatic, anti-inflammatory, cardioprotective [1], hepatoprotective [11], and antigenotoxicity [2] effects of *WC*.

## 2. Methodology

For this review article, the authors searched the literature utilizing three main search engines including PubMed (http://www.ncbi.nlm.nih.gov/pubmed), Google Scholar (http://scholar.google.com), and Science Direct (http://www.sciencedirect.com). This review explores the antilipidemic, antidiabetic, antioxidant, antigenotoxic, hepatoprotective, renoprotective, anticancer, and anti-inflammatory properties of *WC* and its association with human diseases. The references included in this review contain 92 papers, comprising 85 original articles, 5 review articles, and 1 book and 1 congress abstract, dating from 1978 to May 2019. All full texts and abstracts of studies related to *WC* have been taken, among which 2 documents have been written in Spanish and 1 document has been written in Persian, while the rest of the papers are in English. Unpublished congresses, communications, and thesis works have been excluded. The following key search terms were used (“*Nasturtium officinale* L.” OR “*N. officinale* L.” OR “Watercress”) OR (“*Nasturtium officinale*” OR “Watercress” AND “anti-lipidemic” OR “anti-diabetic” OR “antioxidant” OR “anti-genotoxic” OR “hepatoprotective” OR “renoprotective” OR “anti-cancer” OR “anti-inflammatory”). The data taken was confirmed for their suitability and accuracy by the authors. The final data was prepared and has been discussed in the following sections.

### 2.1. Phytochemical Characterization of Watercress

**Figure 2:**
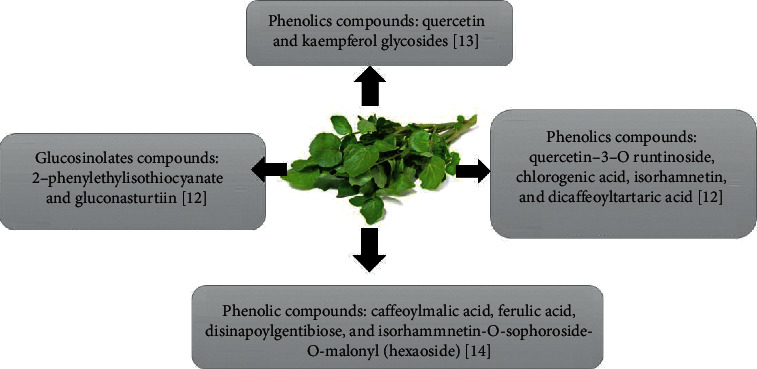
The main phytochemicals of Watercress extract.

Phytochemical characterizations of different parts of *WC* were evaluated with a variety of methods (Figure 2). The high-performance liquid chromatography (HPLC) method showed two main types of metabolites in fresh baby-leaf of *WC* which are phenolics and glucosinolates. As shown in Table 1, the main recognized phenolics were quercetin-3-O rutinoside, chlorogenic acid, isorhamnetin, and dicaffeoyltartaric acid, while the glucosinolates were made up of 2-phenylethyl isothiocyanate (an antimicrobial compound) and gluconasturtiin (the precursor of the anticarcinogenic) [12].

Major phenolics that are found in extracts of *WC* leaves were quercetin and kaempferol glycosides [13]. Another study showed that caffeoylmalic acid was the major phenolic composite in the *WC* extract; nevertheless, other components including ferulic acid, disinapoylgentibiose, and isorhamnetin-O-sophoroside-O-malonyl (hexoside) were detected as minor compounds [14]. Boligon et al. reported higher levels of hydroxycinnamic acids (chlorogenic and caffeic acids) than flavonoids (rutin) in *WC* extract [9]. On the contrary, Aires et al. determined isorhamnetin and rutin as major compounds in *WC* extracts [12]. 8-methylsulphinyloctyl isothiocyanate (MSO) and phenethyl isothiocyanates (PEITC) are two different isothiocyanates derivatives of *WC* [15]. Rose et al. indicated that *WC* extract contains a high concentration of glucosinolates [17] and carotenoids such as lutein and *ß*-carotene [16]. However, another study demonstrated that the aerial parts of *WC* comprise the highest content of total phenolics compounds with an antioxidant activity which could be a valued natural antioxidant [19].

The main components in the oil of *WC* flowers were limonene (43.6%), p-cymene-8-ol (7.6%), *α*-terpinolene (19.7%), and caryophyllene oxide (6.7%). Caryophyllene oxide (37.2%), *α*-terpinolene (15.2%), p-cymene-8-ol (17.6%), and limonene (11.8%) were the central compounds in stems of WC, while myristicin (57.6%), *α*-terpinolene (8.9%), and limonene (6.7%) were the main components in the oil of *WC* leaves [5]. Zeb identified 20 components in the root of *WC* such as the sinapic acid, coumaric acid, and its derivatives, quercetin derivatives, and caftaric acid which were the main phenolic components. Caftaric acid, coumaric acid, and its derivatives and quercetin derivatives were present abundantly in the leaves of *WC* among the 14 phenolic composites. Total phenolic compounds were higher in the methanolic extract of *WC* rather than aqueous extract [18]. In another study, phytochemical analysis has discovered the presence of glucosinolates [20], phenolics, and flavonoids as principal compounds in *WC* extract [21].

*Watercress* contains some vitamins such as B, A, K, E, C, and B_9_, some ions/elements like calcium, magnesium, phosphor, potassium, iron, zinc, and sodium, and some substances like lutein, *ß*-carotene, quercetin, and zeaxanthin [22]. Previous studies confirmed high antioxidant activities of *WC* extracts [9, 13, 14, 18, 19, 23–28].

### 2.2. Effects of Watercress Extract on Lipid and Glucose Levels

**Figure 3:**
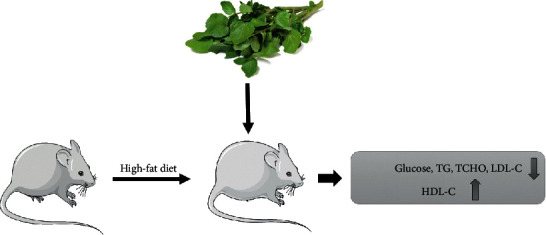
Effects of Watercress extract on lipid and glucose levels.

Herbal medicines are commonly considered to have fewer side effects. They also are less toxic in comparison to synthetic agents. A resurgence of interest in this field resulted in the introduction of new therapeutic compounds including hypoglycemics and hypolipidemic agents [29]. Consuming *WC* extract can reduce the complications of hypercholesterolemia and hyperglycemia. Intragastric administration of *WC* (500 mg/kg BW per day for 10 and 30 days) to rats with hypercholesterolemia lowered their serum total cholesterol (TC) (by 34.2 and 37%), triglycerides (TG) (by 30.1 and 44%), and low-density lipoproteins cholesterol (LDL-C) (by 52.9 and 48%) although it raised the serum high-density lipoproteins cholesterol (HDL-C) level (by 27 and 16%) (Figure 3) [29, 30]. The atherogenic index (AI, determined in terms of LDL/HDL ratio) and the HMG CoA reductase activity showed a significant decrease in rats with hypercholesterolemia which were treated with *WC* in comparison to rats with a high-fat diet [30]. Hadjzadeh et al. showed that in four weeks, using a hydroalcoholic extract of *WC* leaves (200 mg/kg) in treating diabetic rats could remarkably lower levels of TC and LDL-C in comparison to diabetic rats which left untreated but it had no effect on TG and HDL-C levels [31]. Another study observed that the ingestion of raw food (the mixture of 1 portion of *WC* and 1 portion of black rice bran) for four weeks outstandingly lowered TC and TG levels and heightened HDL level but supplementation did not have a significant effect on LDL levels in the experimental animals with diabetes mellitus (DM) [22]. Contrastingly, Gill et al. showed that daily consumption of 85 g raw *WC* for 8 weeks did not have any effect on the plasma lipid profile (TG, TC, HDL-C, and LDL-C) [21].

Hoseini et al. treated diabetic rats with diverse doses of the ethyl acetate (5 to 200 mg/kg) methanol and aqueous extracts (10 to 1000 mg/kg) of *WC* for short-term (7 days) and long-term (56 days) studies with the use of gavage [32]. They observed that after one-week treatment with 800 and 1000 mg/kg of the methanolic extract of *WC* and two months of treatment with 100 mg/kg of the ethyl extract blood glucose level remarkably decreased in comparison to untreated rats which were diabetic [32]. In line with these results, another study showed that the treatment of diabetic rats with hydroalcoholic extract of *WC* (200 mg/kg) leaves over four weeks outstandingly mitigated serum glucose in comparison to diabetic untreated rats [31]. Syamsinah and Anggraini demonstrated that a raw food (the mixture of 1 portion of *WC* and 1 portion of black rice bran) for 4 weeks was able to lower the blood sugar content of the DM experimental animals [22]. Aqueous *WC* extract administration to hyperglycemic rats decreased glucose levels (76.6% higher than insulin) and increased the number of *ß*-cells in the pancreas of treated animals [33]. Fenton-Navarro et al. showed that the hypoglycemic effect of aqueous *WC* extract during acute mode was 76.6% more than that of insulin, and once aqueous *WC* was used in the chronic period, the levels of glucose reached the normal range during the third week to the eighth week of the experiment. [34].

It seems that inhibition of carbohydrate digestive enzyme activity is an effective method of control for hyperglycemia in type 2 diabetes [35–37]. *WC* juice could restrain digestive enzymes in a dose-dependent manner, with a sturdier effect on *α*-glucosidase rather than lipase and *α*-amylase activity. *WC* contains roseoside, pinoresinol, and glycosides, malic acid, and phenolic compounds [14]. The first three of them are *α*-glucosidase inhibitory factors [38, 39], malic acid was recognized as the active agent for hindrance of *α*- amylase, *α*-glucosidase, and lipase in Flacourtia inermis Roxb fruits [35], and the last of them were also identified to interfere with pancreatic lipase activity [35–37]. Spínola et al. suggested that the preventing effects of *WC* juice against digestive enzymes may postpone carbohydrate and lipid hydrolysis in the digestive tract, which consecutively reduces the absorption of fatty acids and glucose [14].

The reduction of TG after the administration of *WC* might be attributed to decreased TG absorption and higher excretion of TG via feces [40]. The decrease in the level of TC after receiving *WC* may be due to increased excretion of bile acids, lower absorption of TC from the intestine, binding of *WC* with bile acids in the intestine [41], reduction of cholesterol biosynthesis [42], and increasing receptors of LDL [31]. Furthermore, other studies suggested that the reduction of TC after treatment with *WC* may be related to valued polyphenolic combinations such as total phenolics and flavonoids in this extract [29, 43].

The decrease in the level of glucose after administration of *WC* extract may be due to its antioxidant properties and stimulating Langerhans islets, improving insulin secretion, and finally reducing blood glucose contents [31].

### 2.3. Effects of Watercress Extract on Antioxidant System

Administration of *WC* extract in hypercholesterolaemic rats markedly augmented the superoxide dismutase (SOD) activity and catalase (CAT) activity and reduced glutathione (GSH) content in liver tissue while it significantly reduced hepatic glutathione reductase (GR) and glutathione peroxidase (GPx) activity as well as malondialdehyde (MDA) level [29]. Gill et al. showed that while the consumption of 85 g of raw *WC* for 8 weeks along with their usual diet significantly increased plasma antioxidants (lutein and *ß*-carotene) in comparison to the control phase, the level of detoxifying enzymes (GPx and SOD) in erythrocytes and plasma total antioxidant status were not changed [21]. Hart showed that treatment with ethanolic extract of *WC* (50–500 mg) inhibited the lipid peroxidation process in different tissues such as the liver (88.78–97.41%), brain (64.66–92.69%), and kidney (71.80–97.06%) [27]. Fogarty et al. showed that exercise-induced oxidative stress increased LPO, H_2_O_2_ accumulation, and lipid hydroperoxides (LOOH) while consumption of acute (2 h before exercise) and chronic (8 weeks) *WC* decreased LPO and H_2_O_2_ accumulation and LOOH formation. In addition, the levels of lipid-soluble antioxidants such as xanthophyll, *γ*-tocopherol, and *α*-tocopherol markedly increased after consumption of *WC* in acute and chronic groups [44]. Zargari et al. claimed that the administration of *WC* extract in arsenic-induced oxidative damage markedly augmented the activities of antioxidant enzymes such as GPx and decreased MDA content in comparison to the normal group [45]. Cyclophosphamide- (CP-) induced oxidative effect increased LPO in RBC, SOD, and CAT activity in the bone marrow and liver tissue, SOD activity, and GSH to GSSG content in RBC while intake of *WC* in doses of 0.5 and 1 g/kg BW for 15 days prior to CP administration reversed all parameters in comparison to relative control groups [46]. These findings are in line with other findings [29] where the treatment with *WC* significantly reduced MDA content in hypercholesterolemic animals.

Bahramikia et al. showed that the addition of Fe^2+^ or ascorbic acid to the liver homogenate markedly augmented reactive oxygen species (ROS) production, protein carbonyl (PCO) formation (as a member of protein oxidation), lipid peroxidation (LPO), and loss of protein-bound sulphydryl (P-SH) groups while the *WC* extract showed inhibitory activity compared to ROS and PCO formation as well as lipid and P-SH oxidation to different degrees [47]. In another study, treatment with *WC* extract (100 and 200 mg/kg) in gentamicin-induced rats not only significantly reduced ROS formation which was parallel to decrease in serum level of blood urea nitrogen, creatinine, pathological changes in kidney tissue, and LPO but also significantly inhibited the increased level of inflammation markers such as nitric oxide (NO) and tumor necrosis factor-alpha (TNF-*α*) [8]. The derivatives of *WC* juice including quercetin and phenethyl isothiocyanates (PEITC) have been shown to induce GST and CYP1A1 activities [48, 49]. Quercetin and PEITC increased transcription of GST and CYP1A1 by binding to xenobiotic responsive element and antioxidant responsive element region of the DNA [50, 51].

Keser et al. reported that lead (Pb) poisoning led to the formation of lipid oxidation and activated antioxidant defense enzymes such as ascorbate peroxidase (APX), SOD, CAT, and GR in leaves of *WC* [6]. APX and CAT are metalloenzymes containing Fe. Srivastava et al. showed that treatment with arsenic decreased Fe concentration in plant tissues [52]. It can be concluded that decreasing activity of CAT and APX in roots of *WC* plants (at high arsenic concentration) is due to a shortage of Fe for the synthesis of these enzymes. Namdjoyan et al. suggested that treatment of *WC* plants with nitric oxide molecule could indirectly or directly induce antioxidant enzymes to tolerate arsenic-induced damage [53] for the reason that NO has an antioxidant function and scavenges ROS that is produced in oxidative stress [54]. Rose et al. demonstrated that PEITC and MSO (the constituents of *WC*) reduced cyclooxygenase-2 (COX-2) and inducible nitric oxide synthase (iNOS) protein expression levels [15]. In vitro exposure of peripheral blood mononuclear cell (PBMC) to WC or PEITC (24 h) augmented the gene expression of GPx and SOD. Additionally, *WC* extract increased SOD activity nearly twofold, while PEITC had no effect [55]. This potential may be due to auxiliary bioactive components in *WC* extract such as hydroxycinnamic acids or quercetin glycosides [56].

The strong antioxidant activity of ethanolic extracts of *WC* has been confirmed by diverse mechanisms including binding to transition metal ions, inhibition of chain beginning, removal of peroxides, scavenging of free radicals, and prevention of continual hydrogen abstraction [27, 57]. Lhoste showed that *WC* could activate phase I and phase II detoxification enzymes' activities and inhibited genotoxicity in HepG2 cells [58]. According to the high total of flavonoids and phenolics of *WC* extract, it has the potential for inhibition of LPO, enhancement of antioxidant capacity, and restoration of GSH content in the animal model with a high-fat diet [29]. There is a phenolic hydroxyl group in polyphenols that competitively reacts with ROS and reduces LPO. Moreover, flavonoid or phenolic compounds of *WC* extract have adequate ability to scavenge NO molecule. Bahramikia et al. observed that the various antioxidant properties of *WC* may be related to direct trapping of ROS, reductive capacity, metal chelating ability, and LPO inhibition [26].

A high concentration of lipid-soluble antioxidants and cellular exposure to constituents of *WC* (such as isothiocyanates) may indicate a probable mechanism for the reduction of LOOH. It is plausible that the elevated lipid-soluble antioxidants can directly scavenge superoxide radicals and lead to a reduction of H_2_O_2_ formation. Gill et al. showed that *WC* can elevate aqueous and lipid-soluble antioxidants in healthy adults as follows: *ß*-carotene (33%), ascorbic acid (35%), and *α*-tocopherol (26%) [21]. Forgarty et al. showed that the lipid-soluble antioxidants including *γ*-tocopherol, *α*-tocopherol, and xanthophyll augmented after consumption of WC [44].

Akbari Bazm et al. investigated the effect of *WC* on oxymetholone-induced oxidative injury in mouse testis. They showed that *WC* extract at 50 and 100 mg/kg dosages meaningfully increased the testis tissue FRAP levels and at a dosage of 100 mg/kg significantly diminished the serum levels of NO. They concluded that *WC* extract has protective effects against testicular toxicity caused by oxymetholone [59]. Our study showed that *WC* extract (500 mg/kg) significantly augmented GPx activity and T-SH level in comparison to acetaminophen- (APAP-) treated rats [11]. In addition, our group reported that *WC* extract markedly increased GPx enzyme activity and significantly diminished hydroxyproline and PCO levels in the liver tissue of bile duct ligated (BDL) rats [60].

### 2.4. Effects of *Watercress* Extract on DNA Damage

**Figure 4:**
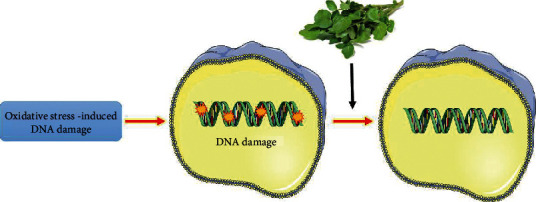
Effects of Watercress extract on DNA damage.

DNA damage has a vital role in cancer, mutagenesis, aging, and other disorders. Consumption of food that is rich in polyphenols such as *WC* extract is a good strategy in reducing oxidative stress and DNA damage (Figure 4) [61]. *WC* extract did not activate cytotoxicity, chromosomal instability, and clastogenicity [62]. In addition, *WC* extract had no role in genetic damage [61] and is not genotoxic in vivo [63].

Casanova et al. showed that aqueous extract of *WC* had an antigenotoxic effect against H_2_O_2_-induced DNA damage [62]. Fogarty et al. showed that exercise-induced oxidative stress increased DNA damage, while consumption of *WC* at acute (2 h before exercise) and chronic (8 weeks) phases attenuated DNA damage [44]. Another study showed that intake of 85 g raw *WC* for 8 weeks daily significantly reduced DNA damage in lymphocytes [21]. Zargari et al. indicated that arsenic-induced oxidative stress increased DNA damage, whereas *WC* consumption significantly decreased 8-hydroxydeoxyguanosine (one of the major products of DNA oxidation) level in liver tissue [45]. Boyd et al. showed that treatment with 50 *μ*l/ml of *WC* extract significantly reduced H_2_O_2_-induced DNA damage in HT29 cells [56]. Another study showed that the *WC* extract exerted antigenotoxic activity against oxidative damage after 4 hours of incubation [64]. In addition, a *WC*-supplemented diet demonstrated a protecting effect against in vivo DNA damage induced by CP [63].

A high concentration of lipid-soluble antioxidants and cellular exposure to components of *WC* (antioxidants and isothiocyanates) may show a potential mechanism for the stabilization of cell DNA [21]. Another study suggested that the antimutagenic effect of *WC* may be related to the antioxidant capability of the extract because leaves of *WC* were proved to have a high content of polyphenols, carotenoids, glucosinolates, and chlorophyll [62]. Hofmann et al. observed that in vitro and in vivo blood cells respond with bioactive materials in *WC* by upregulation of GPx and SOD expression which may be a reason for reducing DNA damage following *WC* consumption [21, 55, 56].

PEITC was found to be genotoxic [50] and was not seen in the crude *WC* extract [56]. However, the antigenotoxic effects could not be attributed to PEITC content [56] and may be related to quercetin and hydroxycinnamic acid (HCA) derivatives that were detected in the crude *WC* extract [65, 66]. The antigenotoxic activity of *WC* may be related to the antioxidant ability [64]. Aksornthong et al. showed that heat could deactivate plant myrosinase and could reduce the antitumor activity of fresh *WC* [67]. Another study found that PEITC and *WC* extract independently control cellular metabolism and cooperatively improve the therapeutic effects of radiotherapy [7].

### 2.5. Effects of *Watercress* Extract on Hepatotoxicity

There was no significant change in the activity of aminotransferase enzymes (AST and ALT) in animals treated with the total extract of *WC* or three fractions at the dose of 175 mg/kg in comparison to control rats. Acetaminophen caused significant hepatocellular damage and elevated serum levels of AST, ALT, and LDH (*p* < 0.001), while treatment with the total extract of *WC* (175 mg/kg) and aqueous fraction (50 mg/kg) significantly decreased these markers. The LD50 value of the petroleum extract was more than 3823 mg/kg, while the other two fractions and total extract of *WC* were nontoxic up to 5734 mg/kg. Acetaminophen-induced histopathological changes of the liver were noticeably reversed after treatment by an aqueous fraction and total extract of *WC* [68]. Zargari et al. indicated that *WC* consumption significantly increased the activities of SOD, CAT, and GPx, while it lowered MDA and 8-hydroxydeoxyguanosine (8-OHdG) levels in the liver. They concluded that *WC* reduced oxidative damage induced by arsenic in the liver [45]. Ebadollahi Natanzi et al. evaluated hepatoprotective effects of *WC* by using a rat liver perfusion system. Adult male rats were selected for investigating the hepatoprotective effect of *WC* against acetaminophen toxicity. Alcoholic extract of *WC* significantly lowered the increased activity of both AST and ALT enzymes induced by acetaminophen [69]. Karami et al. examined the hepatoprotective activity of the methanolic fraction of *WC* against gamma radiation-induced hepatotoxicity. They showed that after a pretreatment step with 100 mg/kg BW for 15 days consecutively and 2 h before *γ*-radiation, the frequency of inflammation was significantly lowered. Moreover, a slight decrease in liver cell necrosis, edema, and congestion was observed. The existence of isothiocyanates and phenols in the methanolic fraction of *WC* which acted as antioxidant agents may be an explanation for this protective activity [70]. Recently, in our study, we investigated the hepatoprotective effect of *WC* extract in APAP-induced hepatotoxicity. We showed that administration of *WC* extract (500 mg/kg) caused a marked decrease in AST activity in comparison to APAP treated rats [11]. Sadeghi et al. indicated that *WC* extract reduced liver damage in BDL animals by decreasing the histopathological indices and hydroxyproline level. They recommended that this extract may be a new therapeutic agent for cholestatic hepatic damage [60].

### 2.6. Anticancer Effects of *Watercress* Extract

*Watercress* has antitumor activity since it can interfere with several axes including oxidative stress, apoptosis, cell cycle progression, and MAPK. [21, 71]. Several studies proposed that diets rich in cruciferous vegetable (CV) plants can reduce the risk of lung cancer, colorectal carcinoma, and prostate cancer [72–75]. CVs are rich in glucosinolates and this compound altered to isothiocyanates (ITCs) in the human intestinal microflora or plants [72]. ITCs are characterized as a type of chemopreventive factor which may have a key role in the control of carcinogenesis. It has been shown that ITCs have strong tumor protective effects in experimental models [6, 76]. Morse et al. showed that dietary ITCs prevent the growth of lung tumors in the animal model induced by tobacco-derived carcinogens [77].

Several studies have explored the potential anticancer effects of *WC* extract, most of which mainly focused on its chemopreventive abilities. Boyd and his colleagues proved that DNA injury induced by H_2_O_2_, 4-Hydroxy Nonenal (4-HNE), and fecal water was prevented in colon cancer HT29 cell line when treated by *WC* extract. Moreover, the addition of *WC* extract to hepatic cell line hepg2 has been shown to protect against DNA damage induced by benzo(a)pyrene [56]. Rose et al. revealed that adding 0.5 *μ*M of 8-methylthiooctyl-ITC and 0.2 *μ*M of 7-methylthioheptyl-ITC increased quinone reductase (QR) twofold activity while 5 *μ*M of PEITC was required to achieve the same outcomes. It has been shown that glutathione conjugation of PEITC and S-(N-*β*-phenylethylthiocarbomyl) glutathione (PECG) have the same potency as PEITC in increasing QR activity [17]. Human studies also indicated these chemopreventive capabilities. Hecht et al. stated that stimulation of 4-(methylnitrosamino)-1-(3-pyridyl-1-butanone (NNK), which is an important tobacco carcinogen in smoker participants, was inhibited by the consumption of 56.8 g *WC* for 3 days [78].

Some researchers started to explore the antimetastatic and antiproliferation effect of *WC* [56, 79]. Boyd et al. proved that *WC* extract inhibited the cell cycle progression of HT29 cells and human colon adenocarcinoma cell line. It also inhibited the invasion of the HT115 cell line through matrigel (11). Rose et al. demonstrated that treatment with 7-methylsulphinylheptyl isothiocyanate (a component of *WC*) reduced MMP9 activity in the MDA-MB-231 (human breast cancer cell line). MMP9 is an enzyme with a proteolytic activity that is crucial for cell invasion and usually augmented in cancer cells. Because of the instability of the other ITCs existing in *WC*, Rose et al. did not test them in this investigation but they evaluated broccoli extract and demonstrated that 4-methylsulphinylbutyl (sulforaphane) components of extract had similar anti-invasive and antimetalloproteinase activity to methylsulfinylheptyl-ITC in *WC* indicating that this effect is not limited to specific ITCs [79]. Souza et al. explored the effects of aqueous extract of *WC* on the growth of the Ehrlich tumor. They showed that the animals in the treatment group showed suppression of tumor growth and a small area of necrosis [80]. Moradi and colleagues revealed that with an increase in the concentration of *WC* extract, the survival rate of the cancerous Hela cells was reduced. They concluded that hydroalcoholic fraction of *WC* can prevent the growth of Hela cells and may be considered as an alternative cure for cervical cancer [81].

As mentioned above, *WC* contains abundant amounts of carotenoids such as beta-carotene and lutein. It has been demonstrated that carotenoids are correlated with antitumor activity [82]. Lutein is a powerful antioxidant that inhibits tissue injury and is associated with a decreased risk of colon cancer [83]. Beta-carotene consumed in physiological contents in combination with antioxidant components may have an anticancer effect in healthy populations [84, 85].

### 2.7. Effects of *Watercress* Extract on Nephrotoxicity

Shahani et al. examined the protective effect of hydroalcoholic extract of *WC* against mitochondrial dysfunction induced by gentamicin (GM) in mitochondria of rat's kidney. They showed that administration of GM led to a significant decrease in mitochondrial function and glutathione content in mitochondria isolated from the kidney. They applied hydroalcoholic extract of *WC* to rats treated with GM. They reported that this extract significantly lowered mitochondrial function and glutathione content in the kidney-isolated mitochondria. In comparison to the control group, *WC* extract significantly lowered glutathione oxidation, MDA, and mitochondrial swelling in a dose-dependent manner [86]. Shahani and colleagues in another study showed that administration of *WC* extract (100 and 200 mg/kg) significantly reduced the ROS formation and serum level of blood urea nitrogen, and creatinine modulated the pathological changes in kidney tissue. It also inhibited the elevated level of inflammation markers such as TNF-*α* and NO in GM-induced nephrotoxicity [8]. Mehrabi et al. revealed that there were no substantial effects of *WC* extract administration in urinary and chemical parameters in calcium oxalate crystal formation. However, an extract with a low dosage had some suppressive effects on the formation of kidney stones induced by ethylene glycol in rats [87]. Traditionally, native inhabitants of the Zagros Mountains used the aerial parts of the *WC* for the alleviation of rheumatic pain, abdominal pain, and urinary stones. Furthermore, new studies showed the analgesic activity of *WC* [88, 89]. Zarfari et al. indicated that *WC* extract significantly increased the activity of antioxidant enzymes and total antioxidant capacity, reduced MDA and uric acid levels, and consequently led to a reduction of arsenite-induced renal toxicity [45]. Karami et al. revealed that vancomycin-induced nephrotoxicity increased serum uric acid, creatinine, and MDA in blood and kidney. They showed that after administration of *WC* extract (500 mg/kg), MDA, creatinine, and uric acid levels in serum were significantly reduced [90].

### 2.8. Anti-Inflammatory Effects of *Watercress* Extract

MSO and PEITC are two phytochemical components that are present in the *WC* extract. In vitro study showed that nitrite and prostaglandin E2 synthesis was reduced by ITCs and MSO in Raw 264.7 cells that lower the secretion of proinflammatory mediators. Interestingly, both MSO and PEITC reduce COX-2 and iNOS protein expression by deactivation of nuclear factor-*κ*B and stabilization of I*κ*B*α* [15]. Sadeghi et al. investigated the anti-inflammatory activity of *WC* in the topical and systemic form and recommended this extract as an anti-inflammatory factor [91]. Furthermore, Shahani et al. indicated that treatment with extract of *WC* (50, 100, and 200 mg/kg BW) significantly reduced TNF-*α* and NO in GM-induced nephrotoxicity [8]. Camponogara et al. evaluated the anti-inflammatory effect of *WC* extract on skin inflammation induced by croton oil in mice. They observed that *WC* extract and *WC* gel inhibited the ear edema and decreased the inflammatory cells infiltration for the acute and chronic model. It also decreased the levels of the proinflammatory cytokines (for acute model). This study demonstrated that *WC* may be a favorable topical, anti-inflammatory compound for treating inflammatory diseases [92].

## 3. Conclusion

**Figure 5:**
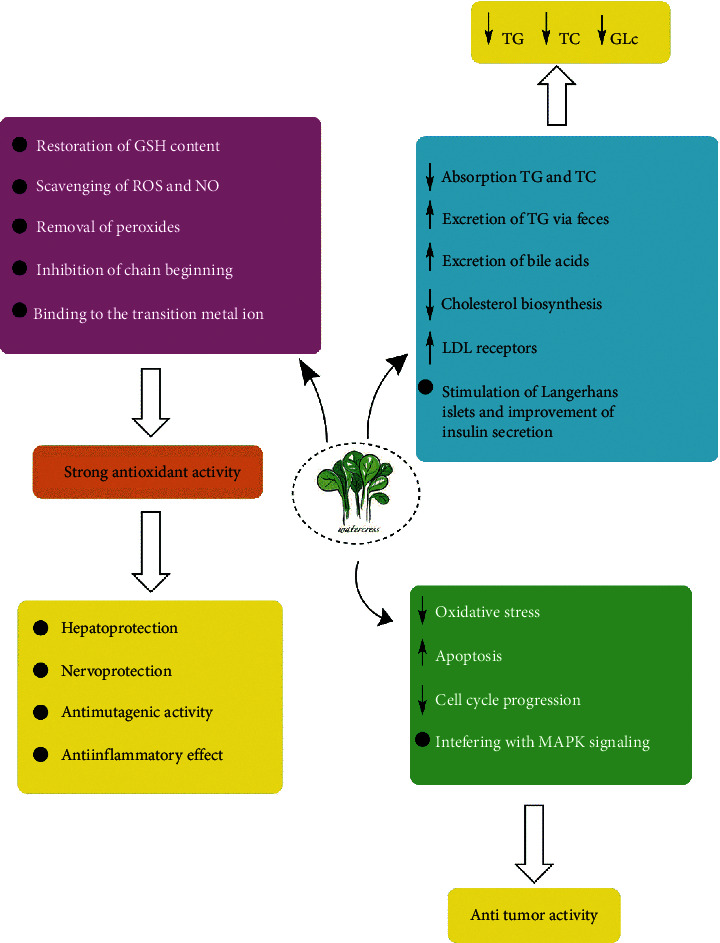
The suggested mechanisms for therapeutic potentials of watercress in human disorders.

Table 2 shows the clinical trials and cell line studies for the *watercress.* In traditional medicine, *WC* is a known remedy for hypercholesterolemia, hyperglycemia, hypertension, arthritis, bronchitis, diuresis, odontalgia, and scurvy. It also acts as an antiestrogenic and can be used as a nutritional supplement. It has been reported that these therapeutic effects are due to primary metabolites such as isothiocyanates, glucosinolates, polyphenols (flavonoids, phenolic acids, and proanthocyanidins), vitamins (B_1_, B_2_, B_3_, B_6_, E, and C), terpenes (including carotenoids), and bioelements which exist in this plant. As shown in Figure 5, the reduction of TG, TC, and glucose after the administration of *WC* might be related to decreased absorption TG and TC, higher excretion of TG via feces, higher excretion of bile acids, reduction of cholesterol biosynthesis, an increase in the numbers of LDL receptors, stimulation of Langerhans islets, and improvement of insulin secretion. The strong antioxidant activity of *WC* extract has been confirmed by diverse mechanisms including binding to the transition metal ion, inhibition of chain beginning, removal of peroxides, scavenging of ROS and NO, and restoration of GSH content. The antimutagenic effect of *WC* may be related to the antioxidant capability of the extract because the leaves of *WC* comprise high content of polyphenols, carotenoids, glucosinolates, and chlorophyll. It has been proved that carotenoids have antitumor activities. Lutein is a powerful antioxidant that inhibits tissue damage and can lower the risk of colon cancer. *WC* has antitumor activity by interfering with several pathways including oxidative stress, apoptosis, cell cycle progression, and MAPK signaling. Furthermore, the anti-inflammatory, hepatoprotective, and renoprotective effects of *WC* extract confirmed that these abilities are related to its antioxidant capacity. Currently, *WC* is the object of numerous pharmacological studies that have demonstrated its antioxidant, anticancer, anti-inflammatory, antipsoriatic, renoprotective, hepatoprotective, and antigenotoxic effects.

## Figures and Tables

**Table 1:** The main recognized metabolites in different parts of *WC*.

Fresh baby-leaf of WC	Phenolics (quercetin-3-O rutinoside, chlorogenic acid, isorhamnetin, and dicaffeoyltartaric acid) [12]
	Glucosinolates (2-phenylethyl isothiocyanate gluconasturtiin)

Extracts of WC	Phenolics (quercetin and kaempferol glycosides) [13] caffeoylmalic acid [14]
	Hydroxycinnamic acids (chlorogenic and caffeic acids) [9]
	Isorhamnetin and rutin [12]
	Isothiocyanates (MSO and PEITC) [15]
	Carotenoids (lutein and *ß*-carotene) [16]
	0047lucosinolates [17]

Oil of WC flowers	limonene, p-cymene-8-ol, *α*-terpinolene, and caryophyllene oxide [5]
Stems of WC	Caryophyllene oxide, *α* -terpinolene, p-cymene-8-ol, and limonene [5]
Oil of WC leaves	Myristicin, *α*-terpinolene, and limonene [5]
Root of WC	Sinapic acid, coumaric acid, and its derivatives, quercetin derivatives, and caftaric acid [18]
Leaves of WC	Caftaric acid, coumaric acid, and its derivatives and quercetin derivatives [18]

**Table 2:** The clinical trials and cell line studies for *watercress*.

Gill CI et al.	A single-blind, randomized, crossover study	85 g raw watercress daily for 8 week	WC supplementation reduces lymphocyte DNA damage and alters blood antioxidant status [21]

Fogarty et al.	Randomized controlled investigation	Acute (consumption 2 h before exercise) and chronic (8 weeks consumption)	Acute and chronic WC supplementation attenuates exercise-induced peripheral mononuclear cell DNA damage and lipid peroxidation [44]

Hofmann et al.	—	85 g WC per day for 8 weeks	WC modulated the enzymes SOD and GPX in blood cells in vitro and in vivo [55]

Boyd et al.	Human colon cancer cells (HT29)	—	WC extract protective against the three stages of the carcinogenesis process [56]

Lhoste et al.	Human HepG2 cells	—	WC acted as a bifunctional inducer by enhancing both phase I and phase II enzymes [58]

Hecht et al.	—	2 ounces (56.8 g) of watercress at each meal for 3 days	Effects of watercress consumption on metabolism of a tobacco-specific lung carcinogen in smokers [78]

Rose et al.	Human MDA-MB-231 breast cancer cells	—	WC suppressed matrix metalloproteinase-9 activity and invasiveness of human MDA-MB-231 breast cancer cells [79]

## Data Availability

The data supporting the findings of this study are included within the article.

## Abbreviations

WC: *Watercress*
HPLC: High-performance liquid chromatography
MSO: 8-methylsulphinyloctyl isothiocyanate
TC: Total cholesterol
TG: Triglycerides
LDL-C: Low-density lipoproteins cholesterol
HDL-C: High-density lipoproteins cholesterol
DM: Diabetes mellitus
SOD: Superoxide dismutase
CAT: Catalase
GSH: Reduced glutathione
GR: Glutathione reductase
GPx: Glutathione peroxidase
MDA: Malondialdehyde
LOOH: Lipid hydroperoxides
CP: Cyclophosphamide
ROS: Reactive oxygen species
PCO: Protein carbonyl
LPO: Lipid peroxidation
P-SH: Protein-bound sulphydryl
NO: Nitric oxide
TNF-*α*: Tumor necrosis factor-alpha
PEITC: Phenethyl isothiocyanates
APX: Ascorbate peroxidase
COX-2: Cyclooxygenase-2
iNOS: Inducible nitric oxide synthase
PBMC: Peripheral blood mononuclear cell
BDL: Bile duct ligated
HCAs: Hydroxycinnamic acids
8-OHdG: 8-Hydroxydeoxyguanosine
CV: Cruciferous vegetables
4-HNE: 4-Hydroxy Nonenal
PECG: S-(N-*β*-phenylethylthiocarbomyl) glutathione
GM: Gentamicin.
